# Bipolar Disorder and Parkinson's Disease: A ^123^I-Ioflupane Dopamine Transporter SPECT Study

**DOI:** 10.3389/fneur.2021.652375

**Published:** 2021-04-13

**Authors:** Roberto Erro, Annamaria Landolfi, Giulia D'Agostino, Leonardo Pace, Marina Picillo, Massimo Scarano, Alberto Cuocolo, Sabina Pappatá, Carmine Vitale, Maria Teresa Pellecchia, Palmiero Monteleone, Paolo Barone

**Affiliations:** ^1^Department of Medicine, Surgery and Dentistry “Scuola Medica Salernitana”, University of Salerno, Baronissi, Italy; ^2^Department of Diagnostic Imaging and Radiotherapy, Azienda Ospedaliera Universitaria (AOU) San Giovanni di Dio e Ruggi d'Aragona, Salerno, Italy; ^3^Department of Advanced Biomedical Sciences, Federico II University, Naples, Italy; ^4^Institute of Biostructure and Bioimaging, National Council of Research, Naples, Italy; ^5^Department of Motor Sciences and Wellness, University Parthenope, Naples, Italy

**Keywords:** dopamine transporter, DaTSCAN, lithium, antipsychotics (also called neuroleptics), degeneration

## Abstract

**Objectives:** Bipolar disorder (BD) has been suggested to be a risk factor for the development of Parkinson's disease (PD). Standard treatment of BD includes drugs that are known to induce drug-induced parkinsonism (DIP). Clinical differentiation between PD and DIP is crucial and might be aided by functional neuroimaging of the dopaminergic nigrostriatal pathway.

**Methods:** Twenty consecutive BD patients with parkinsonism were clinically assessed and underwent ^123^I-ioflupane dopamine transporter single-photon emission computer tomography (SPECT). Imaging data of BD patients with pathological scans were further compared to a population of 40 *de novo* PD patients.

**Results:** Four BD patients had abnormal scans, but their clinical features and cumulative exposure to both antipsychotic drugs and lithium were similar to those of BD patients with normal dopamine transporter imaging. BD patients with pathological scans had putaminal binding ratio and putamen-to-caudate ratios higher than those of PD patients despite a similar motor symptom burden.

**Conclusions:** Up to 20% of BD patients with parkinsonism might have an underlying dopaminergic deficit, which would not be due to cumulative exposure to offending drugs and is ostensibly higher than expected in the general population. This supports the evidence that BD represents a risk factor for subsequent development of neurodegenerative parkinsonism, the nature of which needs to be elucidated.

## Introduction

Bipolar disorder (BD) is a chronic mood disorder with onset in early adulthood, and it is characterized by recurrent episodes of mania (BD type 1), hypomania (BD type 2), and depression (1, 2). Parkinson's Disease (PD) is one of the commonest neurodegenerative diseases, with a prevalence of 1% in the population aged > 60 years (3). A recent meta-analysis has suggested that BD would be associated with later development of PD, with a 3-fold higher risk than the general population (4). However, a subgroup analysis showed a greater likelihood of PD diagnosis in studies with shorter follow-up, suggesting a possible misdiagnosis of PD (4). BD treatment includes lithium, antipsychotics, and antiepileptic drugs (AEDs), all of which can cause drug-induced parkinsonism (DIP) (5); thus, overestimation of the presence of PD in BD could have been possible since the individual studies included in the meta-analysis might not have differentiated PD from DIP (6).

In this context, functional neuroimaging of the dopaminergic nigrostriatal pathway is of particular interest. ^123^I-Ioflupane dopamine transporter (DaT) single-photon emission computer tomography (SPECT), i.e., DaTSCAN, is the only approved imaging modality for the evaluation of PD. At variance with DIP, PD presents with a significantly reduced DaT striatal specific binding ratio (SBR), which is indicative of nigrostriatal degeneration (7).

In the current study, we primarily aimed to explore the functional integrity of the dopaminergic nigrostriatal pathway with the use of DaTSCAN in consecutive BD patients with parkinsonism. We further explored the pattern of dopaminergic deficit in this sample in comparison with a population of *de novo* PD patients.

## Methods

Participants were enrolled from the BD outpatient clinic of the Department of Medicine “Scuola Medica Salernitana,” University of Salerno, Italy. The study protocol (no. 91/0716) was approved by the local ethics committee, and written informed consent was obtained from all participants. The study protocol strictly adhered to the guidelines outlined in the Declaration of Helsinki, fourth edition (8).

Consecutive patients were eligible for inclusion if they had a BD diagnosis according to the DSM-5 criteria (9) and a parkinsonian syndrome according to the Movement Disorders Society (MDS) criteria (10). The presence of comorbid psychiatric disorders, such as psychotic disorders, anxiety disorders, and eating disorders, was an exclusion criterion. All patients were euthymic at study inclusion as assessed by total scores of ≤8 at the Young Mania Rating Scale (11) and ≤6 at Montgomery Asberg Depression Rating Scale (12). They were evaluated to gather the following data: age at BD onset, BD type, family history for psychiatric and neurological disorders, and pharmacological status. Specifically, chlorpromazine equivalent doses (CEDs) were calculated (13, 14) and were used to estimate the cumulative exposure of antipsychotic drugs in dose per year as previously suggested (13)—the definition of one dose per year being equivalent to taking 100 mg chlorpromazine daily for 1 year. Similarly, we calculated the cumulative exposure of lithium in dose per year, the definition of 1 _Lithium_dose/year being equivalent to taking 450 mg lithium daily for 1 year.

Motor symptom duration and total score of the MDS–Unified Parkinson's Disease Rating Scale (MDS–UPDRS), part III, were collected, and we further computed lateralized MDS–UPDRS-III subscores for the most affected (MA) and least affected (LA) body side to calculate the clinical asymmetry index [(CAI) = (MA – LA)/(MA + LA), where lower CAI values indicated a decrease of the degree of clinical asymmetry; i.e., CAI = 0 when MAS = LAS) (15). Additionally, we explored the presence of two of the most common and specific non-motor symptoms of PD, namely, the presence of self-reported hyposmia and REM behavioral disorders (RBD), by using a cut-off of ≥6 for the RBD screening questionnaire (16).

### SPECT Studies

All subjects received an intravenous injection of 185 MBq of ^123^I-FP-CIT (DaTSCAN, GE Healthcare) after thyroid block with oral administration of Lugol solution. SPECT studies were performed using a dual-head system equipped with low-energy high-resolution collimators (e.cam, Siemens Medical systems, USA). The acquisition started between 3.45 and 4.15 h after the radiotracer injection and lasted 40 min (17). Images were acquired with a 128 × 128 matrix (zoom: 1.23; pixel size: 3.90 × 3.90 mm), reconstructed using a Butterworth filter (cut-off 0.5, order 10) and corrected for attenuation using Chang's algorithm (μ = 0.06 cm^−1^).

After acquisition, images were analyzed with DaTQUANT (GE Healthcare), a software that uses a predefined voxel-of-interest (VOI) template for automatic asymmetry measurements and putamen-to-caudate uptake ratios and that further provides *z* scores of single VOIs (i.e., right and left striata, putamina, and caudates) (18). These values are based on a database of 196 age-matched healthy subjects from the Parkinson Progression Markers Initiative (PPMI) (19). The subjects involved in this study were scanned at the Department of Diagnostic Imaging and Radiotherapy of the University of Salerno, which is an authorized PPMI site and has undergone a program for technical qualification, quality assurance, and ongoing camera quality control, as per protocol guidelines (19). We used a striatal SBR *z* score of <-2 (20) to classify BD patients with dopaminergic deficits (BD+), whereas SBR *z* scores of −2 or more were deemed normal and used to classify patients as BD– (i.e., without dopaminergic deficits).

### Statistical Analysis

BD+ and BD– patients were compared in terms of all gathered demographic and clinical data by means of the *t*-test for continuous variables and the chi-square or Fisher test for categorical variables, as appropriate, with *p* < 0.05 being deemed significant.

SPECT data of BD+ patients were further compared to those obtained from a population of 40 newly diagnosed, drug-naïve PD patients (i.e., patients who were never exposed to either dopaminergic replacement treatment or any other drugs acting on the central nervous system), whose clinical diagnosis was confirmed over a follow-up period of 6 years from disease onset (21). These patients underwent at the time of the diagnosis the ^123^I-ioflupane DaT SPECT as well as an extensive evaluation including both motor and non-motor assessments, as described in detail elsewhere (22, 23). Although up to about 50% of these patients reported mood dysfunction including depressive and anxiety symptoms (24), none had a formal diagnosis of BD, major depressive disorder, and anxiety disorder, as per study protocol. For the aim of the current work, 40 PD patients with an MDS–UPDRS-III total score similar to that of our BD sample were selected, and their SPECT data were compared: thus, *z* scores for MA and LA putamen and caudate SBR, for putamen-to-caudate binding ratios on both MA and LA sides, and for putamen and caudate asymmetry index [calculated similarly to the CAI; i.e., (LA – MA)/(LA + MA), with higher values indicating stronger asymmetry] were compared between groups using the *t*-test, with *p* < 0.05 being deemed significant. Statistical analyses were performed using Stata v.13 (StataCorp LP, College Station, TX, USA).

## Results

**Figure 1:**
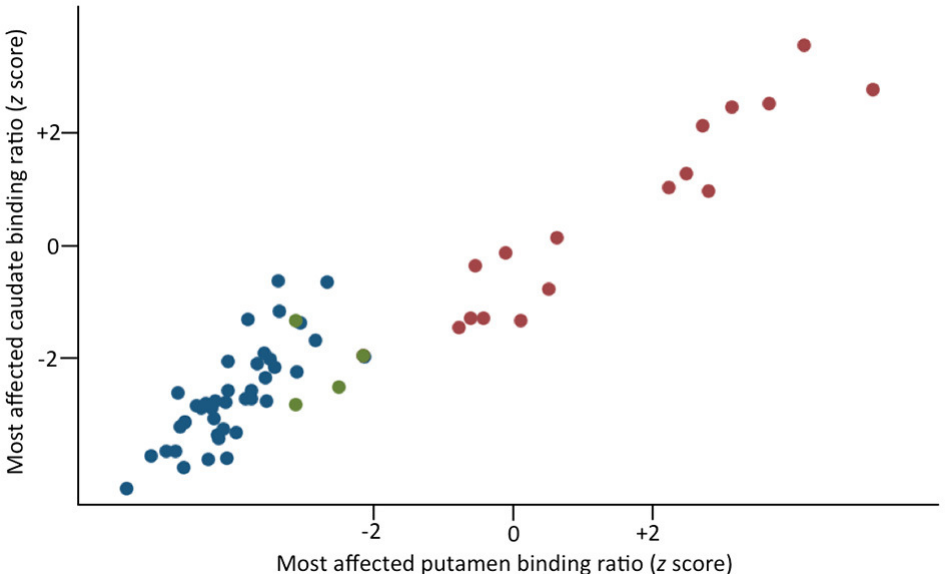
Scatterplot of the binding values (*z* scores) for the most affected putamen and caudate in PD patients (blue circles) and BD patients with (green circles) and without (red circles) dopaminergic deficit.

A total of 20 consecutive patients with BD and parkinsonism (16 men and 4 women) were recruited. Four of them (20%) had abnormal DaTSCAN results and were classified as BD+ (Figure 1). BD+ and BD– patients did not differ in terms of any of the gathered variables (for all, *p* > 0.05; Table 1). Two BD+ patients were started on l-3,4-dihydroxyphenylalanine (l-DOPA) (300 mg/day) and displayed an MDS–UPDRS-III reduction of 12.5 and 35.3%.

**Table 1:** Demographic and clinical comparisons between BD patients with (BD+) and without (BD–) evidence of dopaminergic deficits.

	**BD+**	**BD–**	***p***
Age (years)	72.250 ± 6.898	64.187 ± 9.731	0.147
Sex (M/F)	3/1	13/3	1.000
Age at BD onset (years)	44.250 ± 12.896	31.437 ± 11.057	0.093
Disease duration (years)	28.010 ± 6.143	32.867 ± 12.095	0.505
Family history for psychiatric disorders (yes/no)	2/2	10/6	1.000
Family history for neurological disorders (yes/no)	3/1	14/2	0.509
Polarity at disease onset (depression/mania/hypomania)	4/0/0	8/4/4	0.419
BD type (type 1/type 2)	2/2	13/3	0.249
BD course (IRR/DMI/MDI)	1/2/1	11/5/0	0.112
Lithium (yes/no)	0/4	6/10	0.267
_Lithium_dose/year	3.916 ± 5.5	11.175 ± 25.480	0.585
Antipsychotic drugs (yes/no)	2/2	11/5	0.587
CED (mg/day)	113.090 ± 34.839	137.163 ± 99.271	0.664
_CPZ_dose/year	3.573 ± 4.460	3.936 ± 5.513	0.904
Antiepileptic drugs (yes/no)	3/1	10/6	1.000
VPA (no. of patients)	3	6	
LTG (no. of patients)	0	4	
CBZ (no. of patients)	0	1	
TPZ (no. of patients)	0	1	
SSRI drugs (yes/no)	3/1	4/12	0.101
Tricyclic antidepressant drugs (yes/no)	1/3	2/14	0.509
MDS–UPDRS-III total score	19.501 ± 4.654	23.333 ± 12.871	0.572
Clinical asymmetry index	0.213 ± 0.212	0.194 ± 0.169	0.845
Hyposmia (yes/no)	0/4	5/11	0.530
Smoking history (yes/no)	2/2	4/12	0.549
RBD (yes/no)	1/3	1/15	0.368

*Continuous data are expressed as mean ± standard deviation. BD, bipolar disorder; IRR, irregular course; DMI, sequence depression-mania-free interval; MDI, sequence mania-depression-free interval; CED, chlorpromazine equivalent dose; CPZ, chlorpromazine; VPA, valproate; LTG, lamotrigine; CBZ, carbamazepine; TPR, topiramate; SSRI, selective serotonin reuptake inhibitor; MDS–UPDRS, Movement Disorder Society–Unified Parkinson's Disease Rating Scale; RBD, REM behavioral disorder*.

As compared to PD patients, BD+ patients exhibited an older age at evaluation (60.172 ± 7.914 vs. 72.251 ± 6.891 years, respectively, *p* < 0.001) and longer duration of motor symptoms (13.17 ± 7.496 vs. 33.014 ± 11.489 months, respectively, *p* < 0.001). As per study design, the MDS–UPDRS-III total score did not significantly differ between groups (24.250 ± 11.086 vs. 19.536 ± 8.127, BD+ vs. PD, respectively, *p* < 0.001); however, CAI was found to be significantly lower (indicative of less pronounced clinical asymmetry) in BD+ than in PD (0.213 ± 0.212 vs. 0.651 ± 0.322, respectively, *p* = 0.01). There were no differences in terms of the proportion of patients with and without hyposmia (0/4 vs. 10/30, BD+ vs. PD, respectively, Fisher's *p* = 0.593) and with and without RBD (1/3 vs. 14/26, BD+ vs. PD, respectively, Fisher's *p* = 0.579). Table 2 details the DaTSCAN findings in the two groups. Briefly, BD+ patients had higher SBR in both the MA and LA putamina as well as higher putamen-to-caudate ratios in both hemispheres than had PD patients. Comparisons between BD+ patients and a subgroup of 12 age-matched PD patients confirmed the aforementioned results (Table 3).

**Table 2:** Comparisons of DaT binding values (*z* scores) between BD patients with abnormal DaTSCAN (BD+) and PD patients.

	**BD+**	**PD**	***p***
Most affected putamen	−2.697 ± 0.486	−3.991 ± 0.706	** <0.001**
Least affected putamen	−1.425 ± 1.918	−3.399 ± 0.834	** <0.001**
Putamen binding asymmetry	0.051 ± 0.023	0.114 ± 0.067	0.076
Most affected caudate	−2.167 ± 0.653	−2.673 ± 0.874	0.267
Least affected caudate	−1.795 ± 0.845	−2.159 ± 0.922	0.452
Caudate binding asymmetry	0.051 ± 0.115	0.0738 ± 0.481	0.344
Most affected putamen-to-caudate ratio	0.804 ± 0.194	0.594 ± 0.108	**0.001**
Least affected putamen-to-caudate ratio	0.812 ± 0.218	0.641 ± 0.098	**0.005**

*Data are expressed as mean ± standard deviation; significant p-values are indicated in bold*.

**Table 3:** Comparisons of DaT binding values (*z* scores) between BD patients with abnormal DaTSCAN (BD+) and 12 age-matched PD patients (age = 72.25 ± 6.89 vs. 68.66 ± 1.72, BD+ vs. PD, respectively, *p* = 0.101).

	**BD+**	**PD**	***p***
Most affected putamen	−2.697 ± 0.486	−3.730 ± 0.783	** <0.05**
Least affected putamen	−1.425 ± 1.918	−3.313 ± 1.059	** <0.05**
Putamen binding asymmetry	0.051 ± 0.023	0.121 ± 0.073	0.068
Most affected caudate	−2.167 ± 0.653	−2.607 ± 1.040	0.445
Least affected caudate	−1.795 ± 0.845	−2.174 ± 1.196	0.570
Caudate binding asymmetry	0.051 ± 0.115	0.0708 ± 0.467	0.416
Most affected putamen-to-caudate ratio	0.804 ± 0.194	0.601 ± 0.136	** <0.05**
Least affected putamen-to-caudate ratio	0.812 ± 0.218	0.608 ± 0.117	** <0.05**

*Data are expressed as mean ± standard deviation; significant p-values are indicated in bold*.

## Discussion

In this study, we demonstrated that 20% of BD patients with clinical parkinsonism have an underlying dopaminergic deficit as measured by DaTSCAN. BD+ and BD– patients did not differ in terms of psychiatric or neurological features nor in terms of pharmacological status, implying that differentiation between DIP and neurodegenerative parkinsonism in this population cannot be achieved on a clinical basis alone. This reiterates previous arguments in other psychiatric populations and supports the utility of DaTSCAN for such a differential diagnosis (7, 25).

The question about the nature of this neurodegenerative process in a subset of BD patients remains open, and multiple hypotheses, which are not necessarily mutually exclusive, can be put forward. First, antipsychotic drug exposure has been associated with the development of neurogenerative parkinsonism in an elderly cohort (26), and a neurotoxic effect of lithium has been also postulated (27). However, we found no differences between BD+ and BD– in terms of current and cumulative exposure to both lithium and antipsychotic drugs, which would argue against this hypothesis. A neurotoxic effect on the dopaminergic pathway has been also hypothesized for certain AEDs, especially valproate (28), which was the most commonly used mood stabilizer in both BD+ and BD–. Similar to the above, however, we found no differences between the two groups in terms of AED exposure (Table 1). The possibility remains, however, that neuroleptics, lithium, or AED exposure could have played a role in triggering or fostering a degenerative process in a predisposed subset of BD patients (29). Further research in BD should therefore look at, genetic or otherwise, predisposing factors to the development of an underlying nigrostriatal degeneration.

Two recent studies have recently claimed that BD itself is a strong risk factor for the development of PD (4, 30). However, we found no differences between BD+ and BD– in terms of the clinical features that would be reminiscent of PD, including the presence of hyposmia and RBD. Moreover, the comparison between BD+ and PD patients would further suggest that the two groups might have a different pattern of dopaminergic deficit. BD+ and PD patients had similar MDS–UPDRS-III scores, but the former had a significantly lower clinical asymmetry than the latter, which was not mirrored by a significantly different putaminal SBR asymmetry. Although the latter result might be due to the small sample size, we further note that BD+ patients had higher SBR in the MA putamen compared with PD patients. Altogether, these results would suggest that the motor symptoms in our sample of BD+ patients do not linearly reflect the underlying putaminal denervation, as instead is observed in PD. This discrepancy between the clinical and functional imaging data would suggest that the motor symptoms in BD+ might be due to some extent to the concomitant medications. This might further explain longer motor symptom duration in BD+ than in PD patients (i.e., that they had DIP subsequently “converted” into neurodegenerative parkinsonism), as well as the unsatisfactory response (i.e., <30%) in one of the two patients treated with l-DOPA.

BD+ patients further showed a significantly higher putamen-to-caudate ratio on both sides compared with PD patients. On one hand, this might be interpreted as a relatively lower putaminal involvement in the former and might support the argument raised above that the motor symptoms in BD+ are not entirely attributable to the underlying putaminal denervation. On the other hand, this result might further suggest a higher caudate involvement in BD+. This would implicate caudate dysfunction in the neuropsychiatric features of BD+ and is in line with the evidence linking caudate dysfunction and mood disorders in PD (31, 32).

Apart from dopaminergic dysfunction, which can manifest with different patterns in the nigrostriatal and mesolimbocortical pathways in BD (33) and PD, the two disorders might potentially share the involvement of additional pathophysiological processes. Impairment in the serotoninergic and glutamatergic circuitries has been classically described in BD disorder (34) and is involved in certain motor and non-motor symptoms of PD such as tremor (35) and mood dysfunction (36). Therefore, future studies are warranted to unravel the putative role of other neurotransmitter involvement in BD patients developing degenerative parkinsonism.

Altogether, while our data are consistent with the evidence that a proportion of BD patients might develop degenerative parkinsonism, supporting recent claims (4, 30), the evidence stemming from the comparison with a group of clinically established *de novo* PD patients would raise caution for the interpretation that this neurodegenerative process in BD necessarily reflects PD. Future, well-designed, longitudinal studies should clarify the nature of this degenerative parkinsonism and unravel its pathophysiological underpinnings.

We acknowledge some limitations. First, the small sample size prevents us from drawing firm conclusions about the exact figure of neurodegenerative parkinsonism occurring in BD. Second, we acknowledge that using an SBR *z* score of <-2 might lead to a misclassification error of 2.275% (20). We do not believe this was the case in our sample as the SBR of BD+ patients clearly fell in the range of clinically established PD patients (Figure 1). Moreover, if we were to use a more conservative cutoff of −3 (thus reducing the misclassification error to only 0.135%) (20), two patients (10% of the entire sample) would have been still classified with abnormal DaTSCAN in keeping with neurodegenerative parkinsonism, which is significantly higher than expected in the normal population (3). Third, our BD patients were treated with a number of medications that might interfere with DaTSCAN findings (37). However, no differences in terms of pharmacological status were observed between BD+ and BD– patients. Moreover, previous research has demonstrated a negligible effect of antipsychotic drugs on DaTSCAN quantification, whereas selective serotonin reuptake inhibitors could even increase SBR (37), which might have even led to an underestimation of BD+ in our sample. This might be, however, one reason why 8 (40%) out of 20 BD patients showed SBR *z* score > 2 (Figure 1), indicating DaT overexpression. DaT upregulation, however, has been previously suggested in one study including drug-naïve, euthymic BD patients (38), and we, therefore, cannot claim whether or not what we observed represents a spurious finding owing to the concomitant medications. More generally, previous DaT imaging findings in BD patients (without parkinsonism) yielded controversial results, with some studies suggesting a DaT reduction in BD as compared to that in healthy control (HC) (for a review see (39)). It should be noted, however, that previously reported differences of mean SBR between BD and HC do not necessarily equate to pathological values of DaT uptake as such. A strength of our work is the inclusion of PD patients, by which we demonstrated that SBR in BD+ patients fell within the range of the parkinsonian population, indicative of a degenerative process of the presynaptic dopaminergic terminals.

Age at evaluation was higher in BD+ than in PD patients; however, we performed a subsequent analysis selecting 12 patients from the group of PD patients to match the variable “age” to the BD+ group, which confirmed the aforementioned results (Table 3). Moreover, our data were calculated in contrast to a database of age-matched healthy subjects (18), which indicates that the DaT binding values we measured in the BD+ group can be considered pathological. Finally, if aging was to be the primary factor driving DaT binding loss, one would have expected lower DaT binding ratios than those observed, given the age-related decline in DaT availability (40), which was instead not observed here.

Interestingly, we note that there was a male preponderance in our BD population. Since PD is more common in men (41), we cannot exclude that gender might also play a role in the development of degenerative parkinsonism in BD, an issue that has not been carefully examined in the previous studies (4, 30) and deserves future *ad hoc* investigations, further considering possible different clinical profiles of BD between men and women (42).

Finally, we also acknowledge that the presence of hyposmia and RBD was not checked through formal testing, and this might have led to their underestimation. However, self-reported hyposmia has been shown to sensibly differentiate between DIP and neurodegenerative parkinsonism (25, 43), and the presence of RBD was checked by means of a validated scale in PD (16). Therefore, we do not believe that these limitations have majorly affected our results and their interpretation.

In summary, despite being preliminary, our data show that up to 20% of BD patients with parkinsonism might have underlying dopaminergic deficits, which would support recent evidence that BD represents a strong risk factor for future development of neurodegenerative parkinsonism (4, 30), the nature of which should be, however, clarified. In fact, we cannot claim whether or not the latter reflects true PD, and future larger, long-term studies are warranted to fully elucidate the mechanism behind the link between BD and neurodegenerative parkinsonism.

## Data Availability Statement

The raw data supporting the conclusions of this article will be made available by the authors, without undue reservation.

## Ethics Statement

The studies involving human participants were reviewed and approved by Comitato Etico Campania Sud. The patients/participants provided their written informed consent to participate in this study.

## Author Contributions

RE: conception, data collection, analysis, writing the first draft, and revising the manuscript. AL, GD'A, and LP: data collection, analysis, and revising the manuscript. MP, MS, AC, SP, CV, and MTP: data collection and revising the manuscript. PM and PB: conception, data collection, analysis, and revising the manuscript. All authors contributed to the article and approved the submitted version.

## Conflict of Interest

The authors declare that the research was conducted in the absence of any commercial or financial relationships that could be construed as a potential conflict of interest.

## References

[1] Grande I Berk M Birmaher B Vieta E. Bipolar disorder. Lancet. (2016) 387:1561–72. 10.1016/S0140-6736(15)00241-X 26388529

[2] Murray CJ Lopez AD. Global mortality, disability, and the contribution of risk factors: global burden of disease study. Lancet. (1997) 349:1436–42. 10.1016/S0140-6736(96)07495-8 9164317

[3] de Lau LML Breteler MMB. Epidemiology of Parkinson's disease. Lancet Neurol. (2006) 5:525–35. 10.1016/S1474-4422(06)70471-9 16713924

[4] Faustino PR Duarte GS Chendo I Castro Caldas A Reimão S Fernandes RM. Risk of developing Parkinson disease in bipolar disorder: a systematic review and meta-analysis. JAMA Neurol. (2019) 77, 192–8. 10.1001/jamaneurol.2019.3446 31609378 PMC6802493

[5] Factor SA Burkhard PR Caroff S Friedman JH Marras C Tinazzi M. Recent developments in drug-induced movement disorders: a mixed picture. Lancet Neurol. (2019) 18:880–90. 10.1016/S1474-4422(19)30152-8 31279747

[6] Dols A Lemstra AW. Parkinsonism and bipolar disorder. Bipolar Disord. (2020) 22:413–5. 10.1111/bdi.12888 31954093 PMC7317540

[7] Brigo F Matinella A Erro R Tinazzi M. [^123^I]FP-CIT SPECT (DaTSCAN) may be a useful tool to differentiate between Parkinson's disease and vascular or drug-induced parkinsonisms: a meta-analysis. Eur J Neurol. (2014) 21:1369–e90. 10.1111/ene.12444 24779862

[8] Wolinsky H. The battle of Helsinki: two troublesome paragraphs in the Declaration of Helsinki are causing a furore over medical research ethics. EMBO Rep. (2006) 7:670–2. 10.1038/sj.embor.7400743 16819460 PMC1500825

[9] First MB Williams JB Karg RS Spitzer RL. User's Guide for the SCID-5-CV: Structured Clinical Interview for DSM-5 Disorders, Clinician Version. Arlington, VA: American Psychiatric Association. (2016).

[10] Postuma RB Berg D Stern M Poewe W Olanow CW Oertel W. MDS clinical diagnostic criteria for Parkinson's disease. Mov Disord. (2015) 30:1591–601. 10.1002/mds.26424 26474316

[11] Young RC Biggs JT Ziegler VE Meyer DA. A rating scale for mania: reliability, validity and sensitivity. Br J Psychiatry. (1978) 133:429–35. 10.1192/bjp.133.5.429 728692

[12] Montgomery SA Asberg M. A new depression scale designed to be sensitive to change. Br J Psychiatry. (1979) 134:382–9. 10.1192/bjp.134.4.382 444788

[13] Andreasen NC Pressler M Nopoulos P Miller D Ho B-C. Antipsychotic dose equivalents and dose-years: a standardized method for comparing exposure to different drugs. Biol Psychiatry. (2010) 67:255–62. 10.1016/j.biopsych.2009.08.040 19897178 PMC3677042

[14] Rothe PH Heres S Leucht S. Dose equivalents for second generation long-acting injectable antipsychotics: the minimum effective dose method. Schizophr Res. (2018) 193:23–8. 10.1016/j.schres.2017.07.033 28735640

[15] Plotnik M Giladi N Balash Y Peretz C Hausdorff JM. Is freezing of gait in Parkinson's disease related to asymmetric motor function? Ann Neurol. (2005) 57:656–63. 10.1002/ana.20452 15852404

[16] Stiasny-Kolster K Mayer G Schäfer S Möller JC Heinzel-Gutenbrunner M Oertel WH. The REM sleep behavior disorder screening questionnaire–a new diagnostic instrument. Mov Disord. (2007) 22:2386–93. 10.1002/mds.21740 17894337

[17] Djang DS Janssen MJ Bohnen N Booij J Henderson TA Herholz K. SNM practice guideline for dopamine transporter imaging with 123I-ioflupane SPECT 1.0. J Nucl Med. (2012) 53:154–63. 10.2967/jnumed.111.100784 22159160

[18] Brogley JE. DaTQUANT: the future of diagnosing Parkinson disease. J Nucl Med Technol. (2019) 47:21–6. 10.2967/jnmt.118.222349 30683690

[19] Marek K Chowdhury S Siderowf A Lasch S Coffey CS Caspell-Garcia C. The Parkinson's progression markers initiative (PPMI) - establishing a PD biomarker cohort. Ann Clin Transl Neurol. (2018) 5:1460–77. 10.1002/acn3.644 30564614 PMC6292383

[20] Kreyszig E. Advanced Engineering Mathematics. 4th ed. New York, NY: Wiley (1979). p. 880.

[21] Erro R Picillo M Vitale C Amboni M Moccia M Santangelo G. The non-motor side of the honeymoon period of Parkinson's disease and its relationship with quality of life: a 4-year longitudinal study. Eur J Neurol. (2016) 23:1673–9. 10.1111/ene.13106 27435448

[22] Picillo M Erro R Amboni M Longo K Vitale C Moccia M. Gender differences in non-motor symptoms in early Parkinson's disease: a 2-years follow-up study on previously untreated patients. Parkinsonism Relat Disord. (2014) 20:850–4. 10.1016/j.parkreldis.2014.04.023 24842702

[23] Erro R Picillo M Amboni M Moccia M Vitale C Longo K. Nonmotor predictors for levodopa requirement in *de novo* patients with Parkinson's disease. Mov Disord. (2015) 30:373–8. 10.1002/mds.26076 25648938

[24] Erro R Picillo M Vitale C Amboni M Moccia M Longo K. Non-motor symptoms in early Parkinson's disease: a 2-year follow-up study on previously untreated patients. J Neurol Neurosurg Psychiatry. (2013) 84:14–7. 10.1136/jnnp-2012-303419 22993448

[25] Brigo F Erro R Marangi A Bhatia K Tinazzi M. Differentiating drug-induced parkinsonism from Parkinson's disease: an update on non-motor symptoms and investigations. Parkinsonism Relat Disord. (2014) 20:808–14. 10.1016/j.parkreldis.2014.05.011 24935237

[26] Foubert-Samier A Helmer C Perez F Le Goff M Auriacombe S Elbaz A. Past exposure to neuroleptic drugs and risk of Parkinson disease in an elderly cohort. Neurology. (2012) 79:1615–21. 10.1212/WNL.0b013e31826e25ce 23019267

[27] Lei P Ayton S Appukuttan AT Moon S Duce JA Volitakis I. Lithium suppression of tau induces brain iron accumulation and neurodegeneration. Mol Psychiatry. (2017) 22:396–406. 10.1038/mp.2016.96 27400857

[28] Brugger F Bhatia KP Besag FMC. Valproate-associated parkinsonism: a critical review of the literature. CNS Drugs. (2016) 30:527–40. 10.1007/s40263-016-0341-8 27255404

[29] Erro R Bhatia KP Tinazzi M. Parkinsonism following neuroleptic exposure: a double-hit hypothesis? Mov Disord. (2015) 30:780–5. 10.1002/mds.26209 25801826

[30] Huang M-H Cheng C-M Huang K-L Hsu J-W Bai Y-M Su T-P. Bipolar disorder and risk of Parkinson disease: a nationwide longitudinal study. Neurology. (2019) 92:e2735–42. 10.1212/WNL.0000000000007649 31118242

[31] Weintraub D Newberg AB Cary MS Siderowf AD Moberg PJ Kleiner-Fisman G. Striatal dopamine transporter imaging correlates with anxiety and depression symptoms in Parkinson's disease. J Nucl Med. (2005) 46:227–32. 15695780

[32] Erro R Pappatà S Amboni M Vicidomini C Longo K Santangelo G. Anxiety is associated with striatal dopamine transporter availability in newly diagnosed untreated Parkinson's disease patients. Parkinsonism Relat Disord. (2012) 18:1034–8. 10.1016/j.parkreldis.2012.05.022 22789824

[33] Nikolaus S Mamlins E Hautzel H Müller H-W. Acute anxiety disorder, major depressive disorder, bipolar disorder and schizophrenia are related to different patterns of nigrostriatal and mesolimbic dopamine dysfunction. Rev Neurosci. (2019) 30:381–426. 10.1515/revneuro-2018-0037 30269107

[34] Kato T. Current understanding of bipolar disorder: toward integration of biological basis and treatment strategies. Psychiatry Clin Neurosci. (2019) 73:526–40. 10.1111/pcn.12852 31021488

[35] Helmich RC Dirkx MF. Pathophysiology and management of parkinsonian tremor. Semin Neurol. (2017) 37:127–34. 10.1055/s-0037-1601558 28511253

[36] Barone P. Neurotransmission in Parkinson's disease: beyond dopamine. Eur J Neurol. (2010) 17:364–76. 10.1111/j.1468-1331.2009.02900.x 20050885

[37] Booij J Kemp P. Dopamine transporter imaging with [(123)I]FP-CIT SPECT: potential effects of drugs. Eur J Nucl Med Mol Imaging. (2008) 35:424–38. 10.1007/s00259-007-0621-0 17968545

[38] Chang TT Yeh TL Chiu NT Chen PS Huang HY Yang YK. Higher striatal dopamine transporters in euthymic patients with bipolar disorder: a SPECT study with [Tc] TRODAT-1. Bipolar Disord. (2010) 12:102–6. 10.1111/j.1399-5618.2009.00771.x 20148872

[39] Ashok AH Marques TR Jauhar S Nour MM Goodwin GM Young AH. The dopamine hypothesis of bipolar affective disorder: the state of the art and implications for treatment. Mol Psychiatry. (2017) 22:666–79. 10.1038/mp.2017.16 28289283 PMC5401767

[40] Varrone A Dickson JC Tossici-Bolt L Sera T Asenbaum S Booij J. European multicentre database of healthy controls for [123I]FP-CIT SPECT (ENC-DAT): age-related effects, gender differences and evaluation of different methods of analysis. Eur J Nucl Med Mol Imaging. (2013) 40:213–27. 10.1007/s00259-012-2276-8 23160999

[41] Balestrino R Schapira AHV. Parkinson disease. Eur J Neurol. (2020) 27:27–42. 10.1111/ene.14108 31631455

[42] Diflorio A Jones I. Is sex important? Gender differences in bipolar disorder. Int Rev Psychiatry. (2010) 22:437–52. 10.3109/09540261.2010.514601 21047158

[43] Kim J-S Oh Y-S Kim Y-I Yang D-W Chung Y-A You I-R. Combined use of ^123^I-metaiodobenzylguanidine (MIBG) scintigraphy and dopamine transporter (DAT) positron emission tomography (PET) predicts prognosis in drug-induced Parkinsonism (DIP): a 2-year follow-up study. Arch Gerontol Geriatr. (2013) 56:124–8. 10.1016/j.archger.2012.05.001 22633343

